# Sense of Coherence and Caregivers of Persons with Dementia

**DOI:** 10.3390/bs9020014

**Published:** 2019-01-28

**Authors:** Kristina M. Childers

**Affiliations:** School of Nursing Student in Ph. D Nursing Program, West Virginia University Morgantown, WV 26506-9600, USA; kchilders@mix.wvu.edu

**Keywords:** caregivers (CGs), persons with dementia (PWD), concept analysis, Sense of Coherence (SOC)

## Abstract

Unpaid caregivers (CG) provide most of the assistance to persons with dementias (PWD) living in the community. This study explores the current state of knowledge regarding the concept of sense of coherence (SOC) and CG of PWD via a concept analysis. The identified defining attributes were health, health-related quality of life (HRQoL), CG burden, CG stress, coping as a strength, gender, and decreasing sense of CG coherence over the progression of the disease (dementia). Further study by health care professionals using clinical observations, large samples of respondents, a consistent theory, valid and reliable instruments used to measure defining attributes consistently, and critical reviews of the literature are needed.

## 1. Introduction

Over 16 million Americans are informal (unpaid) caregivers (CGs) to persons with dementia (PWD) [1]. These CGs assist PWDs to overcome a distressing progressive chronic illness, with no cure, treatments that only temporarily minimize symptoms or progression, and many challenges. PWDs experience loss of memory, judgment, communication skills, personality, control of behavior, and even their history of relationship experiences [1]. CGs of PWD may experience impaired psychological and physical health, impaired immune system response, depression, and financial strain while providing almost 18 billion hours of care, and 80% of the overall assistance to PWD [1]. Research indicates there are interventions that improve the quality of life of CGs for PWD, while decreasing emotional stress, improving physical and emotional health, and assisting with caregiving skills [1]. The literature is considerable regarding CGs of PWDs. There is a paucity of research about Sense of Coherence (SOC) and CGs of PWD.

A concept analysis is used to investigate the definition and function of a concept or theory [2]. The purpose of a concept analysis as a research method is to understand the concept of interest and investigate its importance to enhance information and relevance to clinical practice [3]. Antonovsky’s [4,5,6] Theory of Salutogenesis, where one moves towards health along a continuum (illness to health), facing constant change and stress, includes SOC as a main concept. The concept of interest in this analysis is Antonovsky’s SOC [4,5,6], based on the origins of health (salutogenesis) rather than pathogenesis (the origin of disease). SOC [4,5,6] is a way of experiencing the world and challenges via the components of comprehension, manageability, and meaningfulness. Comprehension means the challenge is logical to understand [4,5,6]. Manageability indicates one has resources to cope with the encounter [4,5,6]. Meaningfulness requires that the challenge make sense and be considered to be worthy of effort to respond to stressors or problems [4,5,6]. CGs of PWD are unpaid persons providing care and/or assistance to PWD. 

### 1.1. Concept Analysis

Theorists Walker and Avant [2] advocate nurse researchers describe the facts (a.k.a. phenomena) of a concept using either quantifiable or conversational methods. A concept analysis helps one start to identify how to think rationally about terms and definitions used in theory advancement [2]. The purpose of a concept analysis is to assess the structure and function of the concept of interest and examine how the concept works [2]. Structure indicates the concept is plainly “defined” [2]. Function means the uses of a concept in a theory are “clear” [2]. Persons reading the concept analysis should be able to understand precisely the idea being “described, explained, or predicted” [2]. Analysis of the concept itself is thorough and strict; however the results are not to be interpreted as a “final analysis”, but a snapshot of the concept at the particular moment in time [2]. Concepts are dynamic, with the tentative analysis potentially changing per analyst, time, culture, framework, community, and environmental factors [2]. A concept analysis does encourage interprofessional communication and academic discourse about a concept, prompting ideas as to what it is and is not, and generating methods of measurement. 

### 1.2. SOC 

Antonovsky [4,5,6] identified the salutogenic model after comparing the mental health of female concentration camp survivors to women with no experience of concentration camps in Europe in 1970. The concentration camp survivors were judged to be in relatively stable mental health states, and Antonovsky wondered how these women could survive such stress and tension and remain mentally stable [4,5,6]. The salutogenic theory and SOC concentrates on health and wellbeing (as opposed to illness) as determined by the strength of an individual’s SOC. According to Antonovsky [4,5,6], there are three types of stressors: chronic stressors, major life events, and acute daily hassles. Antonovsky identified chronic stressors (e.g., a lack of knowledge or education, scarcity of resources such as money or a job, isolation from social connections) as the strongest risk to SOC [4,5,6]. Antonovsky [4,5,6] proposed that when faced with a stressor, one might react unreasonably, constructively, or lack any response, depending on how the individual is able to manage conflict. Antonovsky [4,5,6] proposed that a person responds to a stressor by activating generalized resistance resources (GRRs). The individual’s response to stressors and activation of the GRRs depend on the person’s SOC. The GRRs can be financial, emotional, psychological, cultural, or involve social supports or strengths. According to Antonovsky [4,5,6]

a GRR is a physical, biochemical, artifactual-material, cognitive, emotional, valuative-attitudinal, interpersonal-relational, and/or macrosociocultural characteristic of an individual, group, or community that is effective in avoiding or combating a wide variety of stressors and thus preventing tension from being transformed into stress.

SOC is the confidence that one is capable of dealing with life stressors through comprehensibility, manageability, and meaningfulness [4,5,6]. Antonovsky defines comprehensibility as events or happenings that make logical sense, and seem ordered, consistent, and structured, even though the action may not be desirable. Manageability is the extent to which a person feels they can cope based on their resources, including past experiences, social support, and psychological strength [4,5,6]. Meaningfulness is how much one feels the stressful situation makes sense, and how he/she interprets action on the stressor as something worth commitment [4,5,6]. A person’s life experiences are the building materials of SOC. Antonovsky suggested SOC was collectively meaningful across gender, ethnicity, social class, geography, and culture [4,5,6].

Antonovsky identified professionally as a medical sociologist, though researchers in professions of psychology, nursing, social work, nutrition, counseling, and public health have utilized the salutogenic theory and SOC (SOC) concept [7]. SOC is one answer to the question of how some persons are able to remain healthy when experiencing life stressors, while others do not [4,5,6]. The SOC focuses on making order out of disorder and emphasized the importance of coping resources in dealing with stress [4,5,6]. CGs confront chronic stressors while caring for a person with progressive dementia. CGs of PWD may experience an absence of resources (GRRs) and thus have a low SOC [4,5,6], thereby limiting the CG’s ability to positively cope with the caregiving role. The CG must make logical sense (comprehensibility) of the disease process, the care recipient’s changing behaviors, and erosion of the past relationship (spouse, parent, sibling), and assign value to helping the PWD (meaningfulness). In order to help with activities of daily living and meet the needs of the care recipient, and at the same time help the loved one through alien events, the CG must understand the disease process (comprehensibility), know of available resources (comprehensibility and manageability), and effectively manage the use of the reserves. In order to truly care for a loved one with dementia, the CG believes the labor merits the time and emotional, financial, and physical investment (meaningfulness). The SOC concept and salutogenesis can guide health care professionals to strengthen a person’s existing strengths (GRRs) and develop positive ways to manage stress, coping, and health.

The purposes of this concept analysis are to develop further understanding of SOC as it relates to CGs of PWD, and begin to examine the instruments used to measure SOC in CGs PWD.

## 2. Materials and Methods

Walker and Avant [2] identify several reasons to complete a concept analysis including fine-tuning and understanding concepts in a theory, developing a standardized language do describe the concept, developing a new tool, and evaluating existing instruments. There are several methods to complete a concept analysis, but Walker and Avant [2] recommend eight steps as follows:

1. Select a concept.
2. Determine the aims or purposes of analysis.
3. Identify all uses of the concept that one can discover.
4. Determine the defining attributes.
5. Identify a model case.
6. Identify borderline, related, contrary, invented, and illegitimate cases.
7. Identify antecedents and consequences.
8. Define empirical referents.

The steps are not done necessarily in a sequence, but are repetitive, as the analyst continues revising as new understanding evolves during analysis [2]. 

### 2.1. Selecting a Concept

The concept of SOC was chosen because the literature about CGs of PWD includes both negative and positive aspects to the CG role. Antonovsky’s [4,5,6] central question about how some survivors of stressors maintain health while other survivors do not is similar to the question of why some CGs of PWD find joy and meaning in the carer role, and others experience ill-health and a negative quality of life.

### 2.2. Determine the Aims or Purposes of Analysis

The aims of this analysis are to better understand SOC and how it relates to CGs of PWD, and begin to review instruments of measure of SOC and the empirical referents. During the analysis, the researcher must write down the purpose or aims and keep focused [2]. Walker and Avant [2] advise using dictionaries and reviewing the literature to identify as many uses of the concept as possible. The literature review guides the researcher to the defining attributes and evidence for the concept analysis [2].

### 2.3. Identify Uses of the Concept

Walker and Avant [2] guide the researcher to consult dictionaries and the research literature to define and describe the concept. The medical dictionary defines SOC as “a view that recognizes the world as meaningful and predictable” [8]. Merriam Webster’s online dictionary included definitions for the word sense as both a noun and a verb [9]. Sense as a noun [9] is defined as:

“a meaning conveyed or intended, the faculty of perceiving by means of sense organs, conscious awareness or rationality, a particular sensation or kind or quality of sensation, a definite but often vague awareness or impression, a motivating awareness, or a discerning awareness and appreciation”.

Sense as a verb [9] is defined as: “to perceive by the senses; to be or become conscious of; to grasp, comprehend; or to detect automatically especially in response to a physical stimulus (such as light or movement)”. Merriam Webster [9] identifies synonyms of *sense* as “feel, feeling, sensation, perceive, scent, see, smell, or taste”.

Coherence in Merriam Webster’s Dictionary [10] is defined as “the quality or state of cohering: such as systematic or logical connection or consistency or integration of diverse elements, relationships, or values; or the property of being coherent”. Synonyms for *coherence* [10] are: “balance, concinnity, consonance, consonancy, harmony, orchestration, proportion, symmetry, symphony, or unity”.

Antonovsky [4,5,6] defined the concept as:

a global orientation that expresses the extent to which one has a pervasive, enduring though dynamic feeling of confidence that (1) the stimuli from one’s internal and external environments in the course of living are structured, predictable, and explicable; (2) the resources are available to one to meet the demands posed by these stimuli; and (3) these demands are challenges, worthy of investment and engagement.

A review of the literature using the following databases was conducted: Academic Search Complete, Academic Search Premier, Ageline, CINAHL with Full Text, Health Source: Nursing/Academic Edition, MEDLINE, PsycARTICLES, PsycINFO, and Women’s Studies. Keywords that were used were SOC, CG, dementia, and dementia patient. The terms were added into the keyword function and combined using the AND function. Limits to the literature search were CGs of PWD, human subjects, and English language. The date range was January 1979 to August 2018, to evaluate SOC as it relates to CGs of PWD. The studies chosen for inclusion were published from 1994 to 2014 and involved both qualitative and quantitative research methods. The author provides tables of instruments and findings from the literature in the Results section.

### 2.4. Determine the Defining Attributes

Walker and Avant [2] instruct the researcher to try to disclose the collection of attributes most often connected with the concept and that permit the strongest discernment of the concept. Antonovsky [4,5,6] used the terms comprehensibility, manageability, and meaningfulness frequently and routinely. The author expected revisions or additions to the defining attributes following the review of the literature. 

### 2.5. Identify a Model Case

Walker and Avant [2] recommend a researcher find (from the literature or real-world examples) or create a model case, using the best description of the concept, including all the major attributes of the concept. The model case can be simple but assists the researcher to clarify and possibly revise the defining attributes [2]. The researcher uses the model case to understand the concept (SOC of CGs of PWD), clarify understanding, and identify the internal structures of the concept [2].

### 2.6. Identify Borderline, Related, Contrary, Invented, and Illegitimate Cases

The researcher next examines other cases of the concept, which may be comparable or opposite the model case [2]. The borderline, related, contrary, invented, and illegitimate cases [2] allow the researcher to refine the defining attributes and clearly understand what does and does not define the concept [2]. A borderline case [2] covers most but not all of the defining attributes of the concept. A related case [2] again is related to the main concept, but does not contain all the defining attributes, conflicting when carefully investigated. A contrary case [2] is a clear example of “not the concept” (p. 166). An invented case [2] is when the researcher takes the concept outside his or her “own experiences” (p. 166) to examine the relationship to the concept from another point of view. An illegitimate case [2] provides an example of a concept term used inappropriately.

### 2.7. Identify Antecedents and Consequences

According to Walker and Avant [2], antecedents are actions or occurrences in place prior to the existence of the concept. Consequences can occur in response to the concept [2].

### 2.8. Define Empirical Referents

Walker and Avant [2] require the researcher to investigate how the concept might be measured in the final step (empirical referents) of a concept analysis. Empirical referents guide the development of instruments, add to validity and reliability of instruments, and are useful in practice [2]. Empirical referents are not instruments used to measure the concept, but are usually methods of measuring the defining attributes of the concept. For example, the term *considerate* may be an empirical referent of the concept of *caring*.

## 3. Results

For this paper, the operational definition of SOC in CGs of PWD indicates the CG incorporates knowledge of dementia including progression and treatment of the disease, utilizes appropriate and varied coping resources to meet the demands of caregiving, and values the CG role to achieve the highest HRQoL, to decrease burden and stress, and enhance coping skills and strengths, regardless of gender, with the possibility considered the CGs SOC may decrease over the progression of dementia.

### Analysis SOC and CGs of PWD

Walker and Avant [2] describe concepts as the foundation of a theory. Concepts help one organize sensory information, and determine similarities and differences. The words concept and variable are sometimes used interchangeably [2] critical review of the included studies revealed five commonalities of the concept of SOC in CGs of PWD. The first two steps of concept analysis, selecting a concept, and determining the aims or purposes of analysis were completed in the methods section.

#### Identify all Uses of the Concept One can Discover

After reviewing dictionary definitions of SOC (SOC), and the individual terms sense and coherence, the author compiled a list of frequently recurring words. Some of the frequently recurring terms were: comprehensibility, manageability, meaningfulness, GRRs, dynamic and widespread feeling of confidence, structure, sensation, meaning, awareness, logical, balance, and predictable. The following five ideas about SOC and CGs of PWD were identified in the literature: health, health-related quality of life, CG burden and stress, coping and strengths, gender, and decreasing SOC over disease progression. Please see Appendix A for a complete list of terms identified in the search. SOC is a resource promoting health, improving resilience, and leads to more positive mental and physical health, as well as quality of life and wellbeing [11]. Eriksson and Lindstrӧm [12] reported SOC as a resource improving quality of life either directly or with good perceived health as a mediator. The Orientation to Life and SOC scale [5] have been used in over 33 languages, in 32 countries, with multiple cultures (at least 15 different versions of the questionnaire from both Western and Eastern cultures), populations ranging from very young (children) to very old age (adults), in multiple professions, and in groups with multiple disease-specific conditions (rheumatic disease, depression, mental illness, circulatory problems, dementia, etc.) [5,11,12].

##### SOC, Health, and Health-Related Quality of Life (HRQoL)

According to the World Health Organization (WHO), health is not the absence or presence of disease or disability, but a condition including physical, psychological, and social well-being [13]. Researchers define quality of life (QoL) as “multidimensional” wellbeing, including physical wellbeing, psychosocial wellbeing, safety, and self-fulfillment [14] (p. 51) Health-related quality of life (HRQoL) is defined as “an individual’s subjective view of the impact of a health condition on various aspects of his/her well-being” [15] (p. 800) For this concept analysis, the elements of the HRQoL are multidimensional, including physical, psychological, social, and environmental aspects of wellbeing. Two studies specifically addressed HRQoL, while five other studies reported both mental and physical aspects, totaling six research studies [15,16,17,18,19,20,21]. A high HRQoL was predicted by having low distress and being female [15]. SOC was stated to be a strong predictor of quality of life [16]. Ekwall et al. [18] stated a higher mental health quality of life was predicated by a high SOC and using “self-sustaining coping strategies” (outside interests such as work, hobbies, etc.) (p. 592). Mockler et al. [19] screened CGs health for psychiatric morbidity, and reported higher a SOC correlated with lower psychiatric morbidities in CGs. Valimaki et al. [15] reported a significant correlation between SOC and depression (r = −0.632), and distress (r = −0.579) (p. 802), noting depression and distress had significant correlations to SOC and HRQoL. Table 1 shows the measures used, reliability and validity as reported, and associations or findings of SOC, health, and health-related quality of life.

##### SOC and CG Burden and Stress

Eight articles focus on burden or stress as experienced by CGs of PWD living at home [15,16,17,18,19,20]. CG burden incorporates the physical, psychological, socioeconomic, and emotional distress or strain one may experience when caring for another [17,38] described CG burden as the physical and psychological challenges experienced when caring for an ill loved one. Gallagher and colleagues [38] specifically used the term “role overload” as a measure of CG burden, mostly in relation to Pearlin’s model of the stress process.

Potgeiter and Heyns [39] do not specifically define burden or stress, but report CGs of PWD experience psychological burden and mental distress. CGs of persons with Alzheimer’s dementia describe feelings of “anger, loss, social isolation, entrapment, sadness, anxiety, and guilt” [17] (p. 548). Mockler et al. [19] described CGs’ expressed emotion as associated with strain and distress, but did not specifically define stress or burden. Chumbler et al. [40] stated CGs providing care for someone at home may experience stress and exhaustion, both comparable to the definition of burden.

A high QoL was predicted by having low distress and being female [15]. SOC was stated to be a strong predictor of quality of life [10]. The researchers identified above agree that there is burden or stress associated with the CG role. The investigators used different instruments to measure CG burden, role-overload, and/or stress (ors). Table 2 includes measures used, reliability and validity, and associations or findings.

##### SOC and Coping as Strengths

Both Chumbler et al. [40] and Ekwall et al. [18] identified coping as a strength or ability. However, Chumbler et al. [40], Gallagher et al. [38], and Potgieter and Heyns [39] do not define the word coping; both research groups consider effective coping as a measure of SOC, or salutogenesis. It is important to consider that SOC with burden and stress, as well as with coping and strengths, were not measured with separate instruments. The elements were not collapsed together because of the different definitions of burden and stress versus coping and strength. 

Ekwall et al. [18] expressed SOC as a coping strategy. Both Chumbler et al. [40] and Ekwall et al. [18] identified coping as a strength or ability, while Gallagher et al. [38] and Potgieter and Heyns [39] related effective coping as a measure of SOC. Ekwall et al. [18] used Lazarus and Folkman [55] to guide their description of coping, using the concepts of internal and external resources to inform the definition of coping. Internal coping resources depend on the CG relationship to the care recipient, the carer’s personality, and the understanding of the CG role. One may argue that spirituality is an internal coping mechanism. Spirituality in an organized or personal form was one of the most common coping strategies as well as sources of support to rural CGs [56]. CGs who reported using religious coping were more likely to score lower on caregiver burden instruments [57]. External coping resources include the care recipient’s abilities to assist with care, and services such as home health, meal delivery, housekeeping, and the like. There are emotional- and problem-focused coping strategies one uses to solve problems [18]. Ekwall et al. [18] describe emotion-focused tactics as a change to the meaning of the threat or distract a person’s focus from the problematic situation. Problem-focused strategies define the threat and attempt to overcome the issue. The most positive coping methods for CGs were seeking support, remaining future oriented, and religiosity/spirituality [58]. Please see Table 3 for SOC and coping as strengths.

##### SOC and Gender

Seven of the studies focused on gender, although male or female sex is identified in the studies [19,29,38,62]. Chumbler et al. [40] and Ekwall et al. [18] both asked participants to self-identify gender. Pretorius and colleagues [62] used semi-structured interviews, where the data collector could see the respondent to determine gender. Thompson and colleagues [29] used surveys with self-report of gender, but also used bioinstrumentation and blood tests. There were no specific instruments used to determine gender. The research results have varied as to whether women and men experience more, less, or similar burden. Male CGs of spouses with dementia appear to have higher SOC, use more task-oriented problem solving approaches, and manage support resources more effectively than women [29,62]. Thompson et al. [29] suggested men experience less negative effects of caregiving such as depression, anxiety, anger, hostility, and somatic symptoms than women do. Nonetheless, male CGs reported relying on their adult daughters for a great deal of support and help [62].

##### Decreasing SOC over Disease Progression

CGs’ SOC may diminish over time [63]. The researchers reported a decrease in SOC over a three-year progression of dementia. CG SOC was measured using Antonovsky’s SOC scale (29 item) [5]. Depressive symptoms were assessed using Beck’s Depression Inventory (BDI) [64]. The 15D [27] and the Visual Analogue Scale (VAS) for wellbeing [65] measured QoL. Goldberg and Hillier’s [26] General Health Questionnaire (GHQ) was used to measure CG distress. The Clinical Dementia Rating scale (CDR) [66] was used to measure the severity of dementia. Structured interview protocols and scores were used to obtain a sum of boxes scores for the CG interview [67]. Researchers used the inventory to assess activities of daily living for clinical trials in Alzheimer’s disease (ADCS-ADL) CG interviews [65] to evaluate activities of daily life. Finally, the Neuropsychiatric Inventory (NPI) used the CG interview to assess behavioral symptoms of dementia [68].

##### Determining the Defining Attributes

The characteristics of SOC seeming most obvious in defining the attributes were comprehensibility, manageability, and meaningfulness, but after review of the literature, the following attributes were added: health; health-related quality of life; burden/stressful situation; coping as a strength; gender; and decreased SOC over the progression of dementia. 

##### Identify a Model Case

A married couple attends a medical appointment with the primary care provider. The husband and wife discuss her symptoms of forgetting how to follow recipes and paying monthly bills, getting lost when driving in neighborhood of ten years, and difficulty finding words. They receive a diagnosis of dementia. They ask the health care provider for information about dementia symptoms, treatment, and progression. Both are emotional, but holding hands, agree they are a team and have been through wonderful and difficult times throughout their lives. They acknowledge their strength as a couple, support systems in place, and are referred to a dementia support in the local community.

##### Identify Borderline, Related, Contrary, Invented, and Illegitimate Cases

Borderline case. 

A daughter-in-law is caring for her father-in-law who has moved in with her family after a diagnosis of dementia. The health care provider supplied an informational pamphlet about dementia and referred both to a local dementia support network. The father-in-law has health insurance and a pension, but she has four children ranging in age from six years to 14 years. Her children are each in two or three sports or activities, she and her husband both work 40 or more hours per week, and her father-in-law requires more assistance with grooming, bathing, dressing, and meals. She and her husband decide to place her father-in-law in a dementia care facility because they lack the time and energy to continue caring for him at home and know “it is only going to get worse”.

Related case. 

A related case could be about resilience, coping, or stress management. These concepts are related to SOC, but not the same.

Contrary case. 

A daughter discovers her mother has dementia. She “doesn’t want to remember her mother this way”, changes her phone number and moves across the country. 

Invented case. 

A being from another planet arrives on earth and moves into a home of a PWD. The being understands the PWD needs help to take care of the house, prepare meals, and get dressed. The being contacts friends from its planet, and three more beings arrive on earth, move into the home, take six-hour shifts to help their host with dementia, and all live happily ever after.

Illegitimate case. 

A young man uses his five senses of sight, hearing, smell, taste, and touch to determine which restaurant is his favorite. 

##### Identify Antecedents and Consequences

After reviewing the model case and the other cases (borderline, related, contrary, invented, and illegitimate), antecedents and consequences of SOC in CGs of PWD were identified. Antecedents included: the ability to recognize individual interaction with the environment; ability to recognize disorder and change as a normal part of one’s daily life; and the ability to find strategies and resources to cope with chaos as part of daily life. Consequences of SOC in CGs of PWD included an impact on coping skills, quality of life, health (physical and emotional), stress, and burden. 

##### Define Empirical Referents

Empirical referents, according to Walker and Avant [2] have more association with the defining attributes rather than the concept itself. In other words, how do we know if the concept exists in the natural world, and if it does, how might we begin to measure the concept? The defining attributes are comprehensibility, manageability, meaningfulness, health, quality of life, burden or stressful situations, and coping as a strength. Table 4 lists the measurements used for the defining attributes, as well as comprehensibility, manageability, and meaningfulness (SOC). SOC was measured by the SOC scales [5], the 29 item (in eight studies) [15,16,17,19,20,29,38,39] and the 13 item (in three studies), [18,21,46] and with the SOC scale (13 item) in Japanese [47]. Health was measured by the Nottingham Health Profile Scale (NHP) [22], the General Health Questionnaire-28 (GHQ-28) [26], Short Form 36 item (SF-36) [25], EuroQoL Visual Analogue Scale (VAS), and EQ5D [28], two rating scales by authors [21], and the Symptom Questionnaire (SQ) with immunoassays and bioinstrumentation [30,31,32,33,34,35,36,37]. Health-related quality of life was measured in the literature using the EuroQol EQ-5D [23,24], the short version of short form 36 (SF-36) [25], and the 15-D questionnaire and Visual Analogue Scale (VAS) [27]. Burden (CG) was measured with the Caregiver Burden Scale (CB) [41], Sense of Competence Questionnaire (SCQ) [42,43], Role Overload [44,45], the Japanese Zarit Burden Interview (J-ZBI-8) [48,49,50], the Caregiver Burden Index (CBI) [52], and the Screen for Caregiver Burden [53]. Stress was measured by the SOC 29-item scale [5], the General Health Questionnaire (GHQ-28) [26], Greene’s Behavioral Disturbance and Stress Measure (Greene’s Scale) [51], and the Perceived Stress Scale [54]. Coping as a strength was measured by the Carer’s Assessment of Managing Index (CAMI) [55,59], the Coping Resources Inventory [61], the SOC scale (29 item) [5], the Satisfaction with Life Scale (SWLS) [60], the Fortitude Questionnaire (FORQ) [61], and the Three-Dimensional Coping questions [34]. The SOC 29-item scale [5] was used by researchers to assess SOC (comprehensibility, manageability, and meaningfulness, as well as stress in three studies [16,17,21], and in one study [38] as a specific measure of coping. Table 4 includes a list of instruments from the literature review.

Empirical referents [2] are the final step in a concept analysis.

## 4. Discussion

The purposes of the study were to develop further understanding of and begin to examine measures of SOC in CG of PWD. An additional use of a concept analysis is to develop an operational definition [2]. The operational definition of SOC in CGs of PWD indicates the CG incorporates knowledge of dementia including progression and treatment of the disease, utilizes appropriate and varied coping resources to meet the demands of caregiving, and values the CG role to achieve the highest level of health, HRQoL, to decrease burden and stress, and enhance coping skills as strengths, regardless of gender, with the possibility SOC may diminish over the progression of dementia (Childers, 2018).

### 4.1. SOC in CGs of PWD.

Caregiving for a PWD has both positive and negative effects on CGs. Because of the unpredictable behaviors and challenges of the PWD, CGs at times may experience stressors that impact both physical and emotional health. Male and female CGs also experience caregiving, coping, and physical and emotional responses differently [29]. SOC was associated with health, HRQoL, burden and stress levels, role overload, and coping as a strength. CGs routinely participate in the practice of sustaining comprehension, manageability, and meaning.

#### 4.1.1. SOC, Health, and HRQoL

There is a relationship between CG SOC and health [16,17]. The direction of the relationship with mental and physical health and SOC is not clear. Researchers [20] reported high SOC levels are associated with high levels of physical health, but two results of two other studies [18,19] indicate a high SOC is related to high scores in mental health with fewer diagnosable psychiatric disorders, but did not find the same to be true of physical health. The variance in findings can be from many issues, however, the instruments of measure of physical and mental health were varied. 

#### 4.1.2. SOC, CG Burden and Stress

SOC has a negative correlation with burden and CG role overload, indicating those with a higher SOC reported less burden/role overload [16,20,21,38,46]. Gallagher and colleagues [38] found the high SOC score helps adjust to burden by focusing on the meaning of the caregiving experience. CG levels of stress and SOC with the PWD Mini-Mental Status Exam are related to burden scores [21,46]. 

#### 4.1.3. SOC, Coping as a Strength

CGs with a high SOC reported using more positive coping strategies such as managing meaning and learning about dementia, as well as keeping interests outside of caregiving [13,38,44]. Strengths of the CG for coping were support seeking, remaining future oriented, and a belief in a higher power [39]. Potgeiter and Heyns [39] reported CGs who attended group meetings were contributing to the comprehension of the caring experience. Male CGs described coping and strengths as asking for support from their daughters and finding meaning in duties of caregiving [39,44]. CGs who reported a belief in a higher power found meaning and manageability in the caring role [39]. 

#### 4.1.4. SOC, Gender, and Decreasing SOC of the Progression of Dementia

Male CGs reports stressors to include cognitive impairment of spouses with dementia, lack of free time, problem behaviors of the PWD, erosion of the marital relationship, family conflict, and financial worries [44]. Males also reported higher levels of SOC, mental health, social and physical function, and had a higher number of natural killer cell numbers than their female counterparts [29]. Female CGs reported more chronic stress, worry, and fear of incompetence with the caring role [29]. Males tended to manage the carer role as a job or task to be completed, and reported receiving fulfillment when the job was completed [29]. 

One group of researchers described the SOC of CGs may decrease as the disease of dementia progresses [63]. As the demands of the caring role require more effort, the CG’s SOC tended to decrease as the stage of dementia increased. 

#### 4.1.5. Health Care Professionals role in SOC of CGs of PWD

Health care professionals need to recognize the risks of caregiving early and intervene to improve the quality of life for both the CGs and PWD [15]. Health care professions can assess CGs when the PWD attends an appointment or is in the hospital. Early and routine assessment assists to identify potential strains, concerns, dysfunctional coping, and expectations [15,16,17]. A nurse is in the perfect position to offer education, support services, and lead training programs for CGs of PWD. Understanding SOC can guide the coping of CG and positively impact both emotional and physical health. Health care professionals can refer CGs of PWD to local support groups such as the Alzheimer’s Association of the Family CG Alliance National Center on Caregiving. The associations offer CG classes and support groups. The CG may need evaluation for depression and/or anxiety and can be encouraged to speak to a health care provider about the potential benefits and risks of medication, and the benefits of supportive emotional/behavioral/cognitive therapy. The health care professional evaluating and/or treating the PWD can make suggestions for supportive services (home health, home health aide, delivered meals, etc.) as the activities of daily living and instrumental activities of daily living become harder for the PWD. By increasing the GRRs, the health care provider may help increase the CGs SOC. 

### 4.2. Beginning Examination of Measures of SOC in CGs of PWD

The measures for SOC were either the 29-item or 13-item SOC scale [5]. However, the SOC scales were also used to measure coping and stress, in addition to SOC [16,17,21,39]. A variety of instruments were used to measure health, health related quality of life, burden, stress, and coping as strengths. The lack of consistent measures across studies can create problems with correlation, understanding of results, and inconsistent findings. Researchers exploring SOC in the future may use valid and reliable instruments, and measure defining attributes specifically, in addition to SOC.

### 4.3. Advantages and Limitations to Concept Analysis

One advantage of using the Walker and Avant [2] method of concept analysis is the meticulous theoretical and operational definitions for use in research and theory development. Other benefits of concept analysis include clarification in health care jargon or language, and the helpfulness in instrument development [2]. While there are other methods of concept analysis, the steps recommended by Walker and Avant [2] are useful in professions other than nursing, and can be implemented by other health care professionals. 

There are many limitations to concept analysis, including the bias one has when choosing a concept to analyze [2]. The steps are arduous and the analysis can be overwhelming to researchers (particularly novice researchers). Another shortcoming is the impulse to analyze multiple terms and not be able to stop the analysis [2]. Walker and Avant [2] also note there is a fear of sharing the analysis with others, fearing criticism. 

The concept analysis of SOC in CGs of PWD provided a view of this moment in time and is not to be considered a terminal analysis. The study resulted in an operational definition, a clearer understanding of SOC of CGs of PWD, and a brief examination of some instruments used previously to study SOC. 

## Figures and Tables

**Table 1:** Sense of coherence (SOC), health, and health-related quality of life (HRQoL).CG: caregiver; PWD: Persons with Dementia; N/A: not applicable; EuroQoL EQ-5D: European Quality of Life scale.

Measure Com = Comprehensibility Man = Manageability Mean = Meaningfulness	Reference Location Sample Size (*N*)	Instrument	Instrument Reliability and Validity	Instrument Reference	Results—Associations
SOC Com = logical Man = can cope Mean = makes sense	[17] Sweden *N* = 153	SOC scale (29 items) Com = 11 items Man = 10 items Mean = 8 items Choose between 1 (never) and 7 (often)	α = 0.82–0.95 Scores fluctuate between 29 and 203 points Higher = more ability to cope	[5]	Significant association between SOC and Nottingham Health Profile scale (NHP, *p* = 0.000, β = −0.406)
Health CG Nottingham Health Profile scale (NHP)		NHP-38 yes/no items about energy, emotional reactions, social isolation, sleep, pain, and physical mobility 0 = no problem	Not reported	[22]	Significant relation between NHP and total burden, SOC, and age (*p* = 0.000, β = 0.267)
HRQoL		N/A	N/A	N/A	N/A
SOC Com = logical Man = can cope Mean = makes sense	[16] Sweden *N* = 130	SOC scale (29 items) See above	α = 0.82–0.95 Mean score = 151 See above	[5]	Negative association between SOC and age (*r* = −0.19)
Health		NHP scale See above	Not reported	[22]	Burden strongly correlated to NHP (*r* = 0.54, *p* <0.01) NHP significantly related to SOC
HRQoL		EuroQol, EQ-5D Health-related quality based on three levels (no problems; some or moderate problems; and extreme problems or unable), in five domains (mobility, self-care, usual activities, pain or discomfort, and anxiety or depression)	Mean score = 1.38 (SD 1.51)	[23,24]	SOC was a strong predictor of HRQoL
SOC 13-items Com = logical Man = can cope Mean = makes sense	[18] Sweden *N* = 171	SOC scale (13 items) Com = 5 items Man = 4 items Mean = 4 items	Score range 13–91 Mean scores ♂ = 72.1 (SD 12.8) ♀ = 72.9 (SD 13.6) α = 0.85	[5]	
Health		N/A	N/A	N/A	N/A
HRQoL		Short version of SF 36 Short-Form 12 questions about how current health affects life via the mental component summary score (MCS12) and physical summary score (PCS12) Higher score = higher quality of life	Scores MCS12 45.8 Scores PCS12 37	[25]	High scores on SOC predict high scores on MCS12 (*p*-value <0.001), but PCS12 was not significant Asking for social and practical support predicted low quality of life
SOC Com = logical Man = can cope Mean = makes sense	[19] London *N* = 50 CGs living with PWD NSU—nonusers of services SU—users of services	SOC scale (29 items)See above	Mean scores and SD SOC 29 NSU 146.6 (21.6) SU 113.1 (28.4)	[5]	Significant difference between service user group (SU) and non-service user group (NSU) on SOC (*U* = 97.5, *Z* = −3.63, *p* <0.0003) CG in NSU had higher scores on SOC than SU
Health		General Health Questionnaire-28 (GHQ-28) screens for psychiatric morbidity—used for CG health status in study	Mean scores and SD GHQ-28 NSU 9.3 (7.6) SU 15 (12.1) Mean and SD Normative 143 (21) NSU 147 (22) SU 113 (28)	[26]	Did not vary significantly between SU and NSU groups There was an inverse relationship between SOC and GHQ (*r* = −0.46, *p* <0.0006). SOC ↑ as psychiatric morbidity ↓ GHQ-28 scores significantly higher in CG of PWD than normative data indicated
HRQoL		N/A	N/A	N/A	N/A
SOC	[21] Arkansas *N* = 305	SOC scale (13 items)Uses a 7-point scale, possible range from 13 to 91 Higher score = higher SOC	α = 0.86	[5]	Adult children reported lower SOC than spouses or other relatives outside the immediate family
Health		Authors used one self-rating question of how CGs rated their own health considering age and gender 1 = not good at all, 2 = fair, 3 = good, 4 = very good, 5 = perfect CG health also measured by self-reported if diagnosed by health care provider with high blood pressure or hypertension, any heart disease or condition, and trouble with anxiety in the past year.	-- --	None cited in article	CGs reporting unhealthier self-rated health (β = 0.18, *p* <0.001) and problems with anxiety (β = 0.12, *p* = 0.023) experienced higher burden (role overload)
HRQoL		N/A	N/A	N/A	N/A
SOC Com = logical Man = can cope Mean = makes sense	[15] Finland *N* = 170 CG/PWD dyads	SOC scale (29 items)See above	α = 0.001 Factor analysis of SOC resulted in five factors rather than three factors previously reported in literature. Five factors explained 46.6% total variance.	[5]	Women’s SOC significantly lower than men’s (144.2 ± 23.1 vs. 155.7 ± 20.6, *p* ≤0.001) Consistency of life was factor 1, contentment factor 2, purpose factor 3, disappointment factor 4, and interest in life factor 5
Health		No specific measures of health. GHQ 12 item version used not to measure health but to measure extent of distress in this study	α = 0.016	[26]	
HRQOL		15D questionnaire and Visual Analogue Scale (VAS) 15D asks items about mobility, vision, hearing, breathing, sleeping, eating, speech, elimination, usual activities, mental function, discomfort and symptoms, depression, distress, vitality, and sexual activity The VAS assesses overall HRQoL, a 10-cm scale.	α = 0.649 Not reported	[27] Not reported	Statistically significant differences between ♂ and ♀ in dimensions of sleeping and feelings of distress (♀ feel worse). The total amount of drugs used, severe depressive symptoms, and distress were significant predictors of low HRQoL (*R*^2^ = 0.46) Good HRQoL was correlated with strong SOC Depressive symptoms are strongly associated with low HRQoL
SOC	[20] United Kingdom *N* = 170 CGs of PWD	SOC scale (29 items)	α = 0.83 Com α = 0.66 Man α = 0.58 Mean α = 0.61	[5]	
Health		Physical health measured by EuroQoL-Visual Analogue Scale (EQ-VAS) of the EQ-5D—records self-rated health on a vertical VAS, giving measure of health outcome	None reported	[28]	Spousal CGs (mean = 63.24, SD = 13.71) reported higher levels of SOC compared with adult children CGs (mean = 55.97, SD = 11.18, *p* <0.05) SOC positively correlated with physical health
HRQoL		N/A	N/A	N/A	N/A
SOC	[29] New Mexico and Texas *N* = 61	SOC scale (29 items)used as a mediator See above	Mean score 134.5	[5]	Males had higher scores on SOC than females
Health		Short Form 36 (SF-36) 36-item measure developed during Medical Outcomes Study (MOS) Likert scale rating Measure of health concepts across age groups and measures health from respondent’s point of view. Represents eight health concepts: Physical functioning; role limitations due to physical health issues; pain in body; general health; vitality (energy vs. fatigue); social functioning; role limitations due to emotional concerns; and mental health. Also includes self-report of changes in health over past year Symptom Questionnaire (SQ) 92 items in total, 68 are symptoms, 24 are antonyms of some symptoms representing well-being Four scales are depression, anxiety, anger-hostility, and somatic. Each scale is subdivided into symptom subscales and well-being subscales Yes/No responses Respondent describes how he/she feels by selecting yes or no Immune assay and bioinstrumentation monitoring of the relaxation response Immune assays include lymphocytes CD3 (T cell), CD4 (T helper cell), CD8 (T suppressor cell), CD19 (B cell), CD3/HLA-DR (activated T cell), and CD3/CD56/CD16 (NK cell) Bioinstrumentation included: electromyography (EMG), skin conductance, skin temperature, heart rate	Authors report “comprehensive and psychometrically sound” [24] (p. 323) Correlation of changes in split halves of each scale after 2 weeks were: Anxiety = 0.92 Depression = 0.94 Somatic symptoms = 0.86 Hostility = 0.91 Not reported	[25,30,31,32,33,34,35,36,37]	Males reported overall better quality of life Males had higher % of natural killer (NK) cells and lower % of T helper cells than females When Thompson et al. [24] compared NK cell number data to gender and age-matched records using data bank in S. Lewis’s laboratory, no gender differences between male CGs of PWD and non-CGs Female spousal CGs had significantly lower NK cell numbers than female non-CG controls
HRQoL		N/A	N/A	N/A	N/A

**Table 2:** **Sense of Coherence** (SOC) and Caregiver (CG) burden and stress.

Measure Com = Comprehensibility Man = Manageability Mean = Meaningfulness	Reference Location Sample Size (*N*)	Instrument	Instrument Reliability and Validity	Instrument Reference	Results—Associations
SOC Com = logical Man = can cope Mean = makes sense	[17] Sweden *N* = 153	SOC scale (29 items) Com = 11 items Man = 10 items Mean = 8 items Choose between 1 (never) and 7 (often)	α = 0.82–0.95 Scores fluctuate between 29 and 203 points Higher = more ability to cope	[5]	
CG burden		Caregiver Burden Scale (CB) 22 items Indicates CG feelings about PWD Scored from 1 (not at all) to 4 (often) Five indices: General strain—8 items Isolation—3 items Disappointment—5 items Emotional involvement—3 items Environment—3 items	Total Burden Index is mean of all 22 items Higher score = higher burden К-values range from 0.89 to 1 for five indices CB mean score 2.07 in study	[41]	Highest burden was among spouses of PWD 2.4 (Standard Deviation {SD 0.48}, then adult children 2.03 (SD 0.45) Significant associations found between SOC and total burden (*p* = 0.000, β = −0.351)
CG stress		SOC scale (29 items) used to measure ability to manage stressful situations	SOC*—*See above	[5]	Age, gender, and relationship were not related to SOC
SOC Com = logical Man = can cope Mean = makes sense	[16] Sweden *N* = 130	SOC scale (29 items)	α = 0.82–0.95 Mean score = 151 See above	[5]	
CG burden		CB scale See above	CB mean score 2.13 (SD 0.47)	[41]	Highest CG burden identified with spouses (2.48, SD 0.56) and adult children (2.09 (SD 0.47) SOC negatively related to burden (*r* = −0.55, *p* <0.001) Burden and perceived health was influenced by SOC (coping measure in this study). Total burden and SOC (β = −0.330, *p* = 0.000)
CG stress		SOC scale (29 items) used to measure ability to manage stressful situations in the study	α = 0.82−0.95 Mean score = 151	[5]	Negative association noted between SOC and age (*r* = −0.19)
SOC Adaptive coping in study and a measure of stress	[40] Veteran’s Afffairs Medical Centers Florida and Puerto Rico *N* = 102 CG persons with stroke	SOC scale (13-item) short-form version Uses 7-point scale, possible range from 13 to 91 Higher score = Higher SOC	α = 0.86	[5]	Higher SOC associated with fewer depressive symptoms in the study (β = −0.37, *p* <0.0001)
CG burden		Sense of Competence Questionnaire (SCQ) a 27-item instrument measuring level of burden about satisfaction with PWD, own participation as CG, and consequences in own life because of caring for PWD Respondent chooses from 1 (disagree very much) to 4 (agree very much) for each item. The burden score was sum of all item scores. Scores range from 27 to 108, higher score = higher burden	α = 0.89 Mean SCQ score 51.3 (range 29–77)	[42,43]	Higher SOC was associated with lower burden (*p* <0.0001) CGs in study described as low level of burden Negative relationship between SCQ and SOC scores (*r* = −0.53, *p* <0.0001) (higher burden associated with lower SOC)
CG stress SOC used as measure		SOC used to measure ability to manage stressful situations in the study	α = 0.83 prior to study with intraclass correlation coefficient = 0.93) Mean SOC score 71.3 (range 26–91)	[5]	See above
SOC Authors of study defined CG burden as an individual understanding of stress and anxiety (p. 725)	[21] Secondary data analysis Arkansas *N* = 305	SOC scale (13item) See above Used to measure coping	α= 0.75	[5]	Higher SOC associated with lower levels of role overload (measuring burden) Adult children CGs reported lower SOC than spousal CGs and distant relatives (β= −0.05, *p* = 0.52)
CG burden Authors used role overload		Role-overload Four-item scale respondents use to rate levels of overload, consider their individual situations and how they feel. Items measure CG energy level, satisfaction with the care they provide to PWD, and time to complete tasks and care for self 1= not at all, 4= completely	α= 0.73	[44,45]	CG age and race associated with role overload (burden) (β= −0.24, *p* <0.001) indicating CGs who were younger and identified as white, non-Hispanic (β= 0.21, *p* <0.001) had higher role overload (burden) Adult daughters reported more role overload than spouses or distant relatives (β= −0.15, *p* = 0.004) PWD with more impairment in activities of daily living (ADL) and instrumental activities of daily living (IADL) are associated with CG having higher level of role overload
CG Stress		N/A	N/A	N/A	N/A
SOC Used as measure of coping resources in a specific situation	[38] Belgium *N* = 126 CGs of PWD and CGs of persons without dementias	SOC scale (29 item) See above	Mean score 138.16 (SD 21.96) α = 0.84)	[5]	SOC is defensive against CG role overload for CGs of PWD and CG of persons without dementia (in this study indicating a measure of coping in specific situations).
CG burden Role overload used in study		Role overload 4-item scale assessing CG burnout and exhaustion Choices range from never to very often.	Mean 8.67 (SD 3.22) α = 0.72	Pearlin, Mullan, [44]	The association of SOC for CGs of PWD is significant (*r* = −0.56, *p* <0.01) compared to CGs of persons without dementia (*r* = 0.22, *p* ≤0.05) SOC best predictor of role overload for both groups CGs (β = −0.25, *p* = 0.002)
CG Stress		N/A	N/A	N/A	N/A
SOC	[46] Japan *N* = 274	SOC scale (13 item) Choice 7—point scale (1—very often to 7—very seldom or never)	Scores ranged from 13 to 91; higher score = higher SOC Refer to Sakano and Yajima [47] and Andrén and Elmståhl [16]	[5,16,47]	
CG Burden		Japanese Zarit Caregiver Burden Interview (J-ZBI-8)—8 item Japanese version Based on two sub scores—personal strain and role strain Personal strain is related to stress-coping ability (how the individual perceives the stress of situation) Role strain is worry or tension cause by a clash or burden	Refer to Kumamoto et al. [48] and Kumamoto and Arai [49]	[48,49,50]	J-ZBI-8 score was significantly related to the SOC score (*r* = −0.38, *p* <0.001) Both SOC score (β = −0.42, *p* <0.001) and Mini-Mental Status Exam score (β = −0.28, *p* = 0.009) were significantly related to the J-ZBI-8 score (*F*_(2, 76)_ = 10.51, *p* <0.001) Decreased personal strain in J-ZBI-8 was significantly related to a high SOC score (*F*_(3, 75)_ = 8.53, *p* <0.001)
CG Stress		N/A	N/A	N/A	N/A
SOC	[19] London *N* = 50	SOC scale (29 item)		[5]	
CG Burden		N/A	N/A	N/A	N/A
CG Stress		Greene’s Behavioral Disturbance and Stress Measure (Greene’s scale) Used to assess level of CG perceived stress regarding behavior disturbances of PWD Two sections include the behavior or PWD and the stress levels of the CG		[51]	Significant negative relationship between CG SOC scores and CG stress level via Greene’s scale (*r* = −0.38, *p* <0.006) SOC scores increase as stress scores decrease SOC scores varied between service users and non-users of services CG stress levels were positively correlated with psychiatric morbidity (*r* = 0.74, *p* <0.000 with General Health Questionnaire scores
SOC	[39] South Africa *N* = 8 in Qualitative portion, *N* = 6 CGs completing quantitative questionnaires	SOC measured predictability, controllability, and meaningfulness SOC scale (29 item)	Mean score 134.5	[5]	Informal group meeting attendance and support seemed to be most important contributor to comprehension of caregiving experience Manageability was attributed to CGs religion or belief in a higher power, as well as CGs individual abilities helped Religion and/or spirituality was important for CGs to find meaning in the caregiving role
CG burden		Carer Burden Inventory (CBI) Measured CG burden levels relating to PWD behaviors and caregiving situation at home	Mean score CBI 44.63	[52]	CGs reported high SOC and life satisfaction while experiencing similar CG burden and health-related problems
CG stress		General Health Questionnaire (GHQ) Measured CG anxiety, social dysfunction, somatic concerns, and depression	Mean score 12.5	[26]	CGs were identified as being as risk of developing a psychiatric disorder Most health concerns were somatic conditions and anxiety
SOC	[29] New Mexico and Texas *N* = 61 spousal CGs (compared genders)	SOC scale (29-item) Used as mediator See above	Mean score 134.5	[5]	SOC increases opposition to stress (a strong SOC inclines a person to experience life as more secure, predictable, and manageable.
CG burden		Screen for Caregiver Burden A 25-item measure for objective (number of potentially negative experiences) and subjective burden (considered as suffering or stress in response to experiences)	α = 0.88–0.89 internal consistency and test-retest reliability of 0.64–0.70	[53]	No significant gender differences in objective burden scores Significant differences in subjective CG burden scores in response to experiences (considered as suffering or stress) Females reported greater level of burden than males.
CG stress		Perceived Stress Scale (PSS) 14-item instrument measures appraised degree of stress in situations of one’s life. Items measure degree to which respondents perceive life unpredictable, uncontrollable, and overwhelming Rated 5-point scale (0–4). 0 = never, 4 = very often	α = 0.84–0.86 Test–retest stability: 2-day delay 0.85, and 6-week delay 0.55	[54]	Males significantly lower amounts of depression, stress, anxiety, and anger/hostility than females Females reported more emotional stress in measures of depression, stress, and anxiety Males also had lower mental health scores and fewer somatic and total symptoms than females Male NonKiller (NK) cell number was negatively correlated with perceived stress and total symptom score, but no significant correlations among those variables in females

**Table 3:** **Sense of Coherence** (SOC) and coping as strengths. CG: Caregiver; PWD: Person with Dementia

Measure Com = Comprehensibility Man = Manageability Mean = Meaningfulness	Reference Location Sample Size (*N*)	Instrument	Instrument Reliability and Validity	Instrument Reference	Results-Associations
SOC	[18] Sweden *N* = 171	SOC scale (13 item) Com = 5 items Man = 4 items Mean = 4 items	Score range 13–91 Mean Scores ♂ = 72.1 (SD 12.8) ♀ = 72.9 (SD 13.6) α = 0.85	[5]	Having higher quality of life was expected when the CG used self-sustaining coping strategies like having interests outside caring and by high SOC scores
Coping and strengths		Carer’s Assessment of Managing Index (CAMI) Developed to assess individual CGs based on Lazarus’s model of stress and coping 38 items about CGs coping with difficulties in caregiving experience. Based on three themes: problem solving and coping, alternative perception of events, and dealing with stress symptoms Two parts: Part 1—respond to statement being true very often to never Part 2—respond to way of behaving as very helpful to not helpful	Part I CAMI α = 0.86 Part II CAMI α = 0.92	[55,59]	The top five coping strategies after factor analysis were “keeping my emotions tightly under control”, “ ‘taking one day at a time”, “remembering the good times I used to have with the person I care for”, “establishing priorities and concentrating on them”, and “realizing that there is someone worse off than me”
SOC Used as measure of coping resources in a specific situation	[38] Belgium *N* = 126 CGs of PWD and CGs of persons without dementias	SOC scale ( 29 item) See above	Mean score 138.16 (SD 21.96) α = 0.84)	[5]	
Coping and strengths		Three-dimensional coping instruments including: Managing the situation Managing the meaning of situation Managing symptoms of suffering	When three combined into single scale α = 0.69 (three scales individually low reliabilities in sample) Mean = 25.49 (SD 4.96)	[44]	CG with high SOC tend to adjust to burden by focusing on meaning of the caregiving experience. CG with high SOC are less likely to try to manage situations by restricting PWD behavior or by abdicating the caregiving responsibility (CGs of PWD with high SOC tend to use realistic coping approaches) Cognitive coping strategies (managing meaning and learning about dementia) are important to adapt to CG burden for PWD versus persons without dementia
SOC Measure of psychological strength in the study	[39] South Africa *N* = 8 in Qualitative portion, N = 6 CGs completing quantitative questionnaires	SOC scale (29 item) used measured predictability, controllability, and meaningfulness See above	Mean score 134.5 (SD 17.61)	[5]	
Coping and strengths		Psychological strengths were measured by SOC (see above), and two other instruments The Satisfaction with Life Scale (SWLS) [60] measures life satisfaction CGs sense on an intellectual level Scores range from 5 to 35 (5 = low, 35 = high) Fortitude Questionnaire (FORQ) [61] measured how positively CGs considered selves and family, and the level of family support received Scores range from 0 to 80 (lower than 58 = low, higher than 65 = high)	Mean 19.86 (SD 4.98) Mean 52.25 (SD 11.24)	[60,61]	Despite high CG burden and low general health, CGs in study had some satisfaction with lives CGs perceived not receiving a lot of support
SOC	[29] New Mexico and Texas *N* = 61 spousal CGs	SOC scale (29 item) used as a mediator See above	Mean score 134.5	[5]	Males had higher SOC scores than females, suggesting male CGs are more “resilient or hardy” ([24] p. 327)
Coping and strengths		Coping Resources Inventory 60-item tool to measure coping resources in 5 domains. Domains: cognitive (positive self-worth, positive outlook about others, optimism in life); social (supportive social networks); emotional (able to accept and express emotions to ameliorate stress); spiritual/philosophical (religious, family, cultural tradition or personal philosophy); and physical (health promoting behaviors for well-being) 4-point scale	α = 0.89–0.94	[58]	No gender differences in age, length of time being primary CGs of PWD, social support, or coping resources

**Table 4:** Instruments identified in literature review (available psychometrics are included in Table 1, Table 2 and Table 3). SOC: sense of coherence; HRQoL: health-related quality of life; N/A: not applicable; EuroQoL EQ-5D: European Quality of Life scale. ** Distress may or may not be the same as “Stress”.

Reference	SOC	Health	HRQoL	Burden	Stress	Coping and Strengths
[15]	SOC scale (29 item) [5]	N/A	15-D questionnaire and Visual Analogue Scale (VAS) [27]	N/A	General Health Questionnaire-12 (GHQ-12; used to measure distress)**	N/A
[16]	SOC scale (29 item) [5]	Nottingham Health Profile Scale (NHP) [22]	EuroQoL, EQ-5D [23,24]	Caregiver Burden Scale (CB) [41]	SOC scale (29 item) (manage stress) [5]	N/A
[17]	SOC scale (29 item) [5]	NHP [22]	N/A	CB [41]	SOC scale (29 item) (manage stress) [5]	N/A
[18]	SOC scale (13 item) [5]	N/A	Short-version of Short-Form 36 (SF-36) [25]	N/A	N/A	Carer’s Assessment of Managing Index (CAMI) [55,59]
[19]	SOC scale (29 item) [5]	General Health Questionnaire-28 (GHQ-28) [26]	N/A	N/A	Greene’s Behavioral Disturbance and Stress Measure (Greene’s scale) [51]	N/A
[20]	SOC scale (29 item) [5]	EQ-VAS and EQ5D [28]	N/A	N/A	N/A	N/A
[21]	SOC scale (13 item) [5]	Two self-rating questions by study authors [21]	N/A	Sense of Competence Questionnaire [42,43]	N/A	N/A
[29]	SOC 2 scale (29 item) [5]	Short-Form 36 (SF-36) [25] Symptom Questionnaire (SQ), immune assays and bioinstrumentation monitoring of relaxation response [30,31,32,33,34,35,36,37]	N/A	Screen for Caregiver Burden [53]	Perceived Stress Scale (PSS) [54]	Coping Resources Inventory [58]
[38]	SOC scale (29 item) [5]	N/A	N/A	Role Overload [44,45]	N/A	Three-dimensional coping [44]
[39]	SOC scale (29 item) [5]	N/A	N/A	Carer Burden Inventory (CBI) [42,43]	N/A	SOC scale (29 item) [5] Satisfaction with Life Scale [60] Fortitude Questionnaire (FORQ) [61]
[46]	SOC scale (13 item) [5,47]	N/A	N/A	Japanese Zarit Burden Interview (J-ZBI-8) [48,49,50]	N/A	N/A
[63]	SOC scale (29 item) [5]	Beck Depression Inventory	15D measured Quality of Life-not necessarily HRQoL AND GHQ-28 item [26]	N/A	N/A	N/A

## Appendix A

**List A:** Frequently Recurring Terms from Definitions and Review of the Literature including Sense of Coherence (SOC), Sense, and Coherence.

Comprehensibility	Global or world-wide orientation
Manageability	Stimuli from internal and external environments
Meaningfulness	Appreciation
Feeling of Confidence	Perceive by senses
Structure	Logical connection of diverse elements (coherence)
Predictable	Symmetry
Resources are available	Health
Demands	Health Related Quality of Life
Worthy of Investment	Caregiver Burden and Stress
Meaning	Coping and Strengths
Sensation	Gender
Awareness	Decreasing SOC over Disease Progression
Coping	A view of world as meaningful and predictable
Social Support	Generalized Resistance Resources
	Feeling
